# Hematite Nanoparticles-Modified Electrode Based Electrochemical Sensing Platform for Dopamine

**DOI:** 10.1155/2014/396135

**Published:** 2014-07-21

**Authors:** Khosro Zangeneh Kamali, Pandikumar Alagarsamy, Nay Ming Huang, Boon Hoong Ong, Hong Ngee Lim

**Affiliations:** ^1^Low Dimensional Materials Research Centre, Department of Physics, Faculty of Science, University of Malaya, 50603 Kuala Lumpur, Malaysia; ^2^Nanotechnology & Catalysis Research Center, Institute of Graduate Studies, University of Malaya, Level 3, Block A, 50603 Kuala Lumpur, Malaysia; ^3^Department of Chemistry, Faculty of Science, University Putra Malaysia, 43400 Serdang, Selangor, Malaysia

## Abstract

Hematite (*α*-Fe_2_O_3_) nanoparticles were synthesized by the solid transformation of ferrous hydroxide and ferrihydrite in hydrothermal condition. The as-prepared *α*-Fe_2_O_3_ nanoparticles were characterized by UV-vis, PL, XRD, Raman, TEM, AFM, FESEM, and EDX analysis. The experimental results indicated the formation of uniform hematite nanoparticles with an average size of 45 nm and perfect crystallinity. The electrochemical behavior of a GC/*α*-Fe_2_O_3_ electrode was studied using CV and EIS techniques with an electrochemical probe, [Fe(CN)_6_]^3−/4−^ redox couple. The electrocatalytic activity was investigated toward DA oxidation in a phosphate buffer solution (pH 6.8) by varying different experimental parameters. The chronoamperometric study showed a linear response in the range of 0–2 *μ*M with LoD of 1.6 *μ*M for DA. Square wave voltammetry showed a linear response in the range of 0–35 *μ*M with LoD of 236 nM for DA.

## 1. Introduction

Hematite (*α*-Fe_2_O_3_) is thermodynamically the most stable iron oxide with n-type semiconductor properties [1]. Its stability, favorable band-gap (2.1-2.2 eV), and low cost make *α*-Fe_2_O_3_ one of the most promising materials, and it is used in catalysis, electrocatalysis, photocatalysis, electrochemical sensors, and gas sensors [2–5]. The lower band-gap of hematite compared to titanium dioxide enables this material to absorb light in the visible range [6]. The properties of *α*-Fe_2_O_3_ are highly affected by the morphology, crystallite size, and crystallinity of the material. Many synthetic methods for preparing *α*-Fe_2_O_3_ have been investigated. These processes involve the use of a surfactant, very high temperature, corrosive, toxic, or expensive raw materials, or a long synthesizing period [7–9]. Discovering a low-cost and facile method to synthesize *α*-Fe_2_O_3_ with excellent quality and desirable properties has always been a challenge for scientists. In this work, we describe a low-cost, facile method of synthesizing nanosized *α*-Fe_2_O_3_ particles.

Dopamine (DA) is a neurotransmitter that plays an important role in the brain and nervous system. DA is measured in the body using electrochemical sensors for monitoring HIV infection and the diagnosis of Parkinson's disease. The importance of DA in the nervous system has resulted in the fabrication of electrochemical sensors for its detection [6, 7]. Some work has been done to investigate the performance of *α*-Fe_2_O_3_-modified electrode in supercapacitors, but few studies have been done on the electrochemical properties of *α*-Fe_2_O_3_ for electrochemical sensors [4].

The overall objective of the present study was the improvement of the sensitivity toward the electrochemical detection of DA by using an *α*-Fe_2_O_3_ nanoparticle-modified electrode (GC/*α*-Fe_2_O_3_). Initially, *α*-Fe_2_O_3_ nanoparticles were prepared using a simple hydrothermal method and then used to fabricate the sensor electrode. A ferricyanide redox couple was used as a marker to probe the *α*-Fe_2_O_3_ nanoparticle-modified electrode interface with a tunable kinetic barrier. The GC/*α*-Fe_2_O_3_ electrode showed a better electrocatalytic response toward DA oxidation compared to bare GC electrode. The square wave voltammetry and chronoamperometric techniques were also employed for the detection of DA and showed excellent selectivity and sensitivity.

## 2. Experimental Methods

### 2.1. Materials

Chemical reagents such as ferrous chloride tetrahydrate (FeCl_2_ *·*6H_2_O), ferric chloride hexahydrate (FeCl_3_ *·*6H_2_O), dopamine (DA), and ammonia solution (NH_3_) were purchased from Merck and used as received. Sodium dihydrogen phosphate (NaH_2_PO_4_) and disodium hydrogen phosphate (Na_2_HPO_4_) were received from system and used without further purification. All the other chemicals used in this investigation were of analytical grade, unless otherwise stated, and highly pure deionized water was used throughout this experiment. A stock solution of DA was freshly prepared daily by dissolving it into a suitable amount of deionized water prior to use. Electrolyte and phosphate buffer solutions at different pH values were prepared by mixing stock solutions of 0.1 M NaH_2_PO_4_ and 0.1 M Na_2_HPO_4_, and then the required pH values were adjusted by using 0.1 M HCl and 0.1 M NaOH.

### 2.2. Synthesis of *α*-Fe_2_O_3_ Nanoparticles

The *α*-Fe_2_O_3_ nanoparticles were prepared as follows. Typically, 235 mg of FeCl_3_ *·*6H_2_O and 87 mg of FeCl_2_ *·*4H_2_O were dissolved into 300 mL of deionized water and stirred for 10 min. Further, a 25% ammonia solution was dropwise added to the above reaction mixture until the pH of the attained solution was 10. The reaction mixture was vigorously stirred for 1 h at room temperature. The sample was gathered by repeated centrifugation (6000 rpm for 2 min) and washed with 100 mL of deionized water; this procedure was repeated three times, and it was redispersed into the deionized water. Further, the solution was poured into a 50 mL Teflon-lined autoclave and subjected to a hydrothermal process at 180°C for 12 h. Finally, the resulting brownish-orange color precipitate was centrifuged, washed several times with water, and then dried in a hot air oven at 100°C to completely remove the moisture.

### 2.3. Characterization Techniques

The UV-visible absorption spectrum of the *α*-Fe_2_O_3_ nanoparticles in the 190–900 nm spectral range was recorded using a Thermo Scientific Evolution-300 UV-vis absorption spectrophotometer. The PL spectrum was acquired from a Renishaw inVia 2000 system with a laser emitting at 325 nm. The phase identification was recorded by using a Siemens-D5000 X-ray diffractometer with copper K*α* radiation (*λ* = 1.5418 Å) at a scan rate of 0.02°/s. The surface morphology and elemental composition of the *α*-Fe_2_O_3_ nanoparticles were examined using a JEOL field emission scanning electron microscope operated at 10 kV. The surface topography and roughness of the thin film were studied using Agilent's atomic force microscope (AFM) in AC mode. The size and shape of the obtained *α*-Fe_2_O_3_ nanoparticles were studied using a JEOL JEM-2100F high resolution transmission electron microscope.

### 2.4. Electrochemical Studies

An *α*-Fe_2_O_3_ nanoparticle-modified glassy carbon electrode (GC/*α*-Fe_2_O_3_) was fabricated by dispersing 1 mg of the synthesized *α*-Fe_2_O_3_ nanoparticles in 1 mL of deionized water and then sonicating them for 30 min to ensure a homogeneous dispersion. A 5 *μ*L volume of the colloidal *α*-Fe_2_O_3_ solution was cast on a glassy carbon electrode and then dried in a hot air oven at 65°C for 1 h. This GC/*α*-Fe_2_O_3_ NP-modified electrode was used for the electrocatalytic oxidation of dopamine. All the electrochemical measurements were carried out using a VersaSTAT-3 electrochemical analyzer (Princeton Applied Research, USA) with a conventional three-electrode system under a nitrogen atmosphere at room temperature (27°C). The *α*-Fe_2_O_3_ nanoparticle-modified GC electrode, platinum wire, and saturated calomel electrode were used as the working, counter, and reference electrodes, respectively. A 0.1 M phosphate buffer solution (PBS) (pH = 6.8) was used as the supporting electrolyte. All the potentials are quoted against the saturated calomel electrode unless otherwise mentioned.

## 3. Results and Discussion

### 3.1. Optical Properties of *α*-Fe_2_O_3_ Nanoparticles

**Figure 1:**
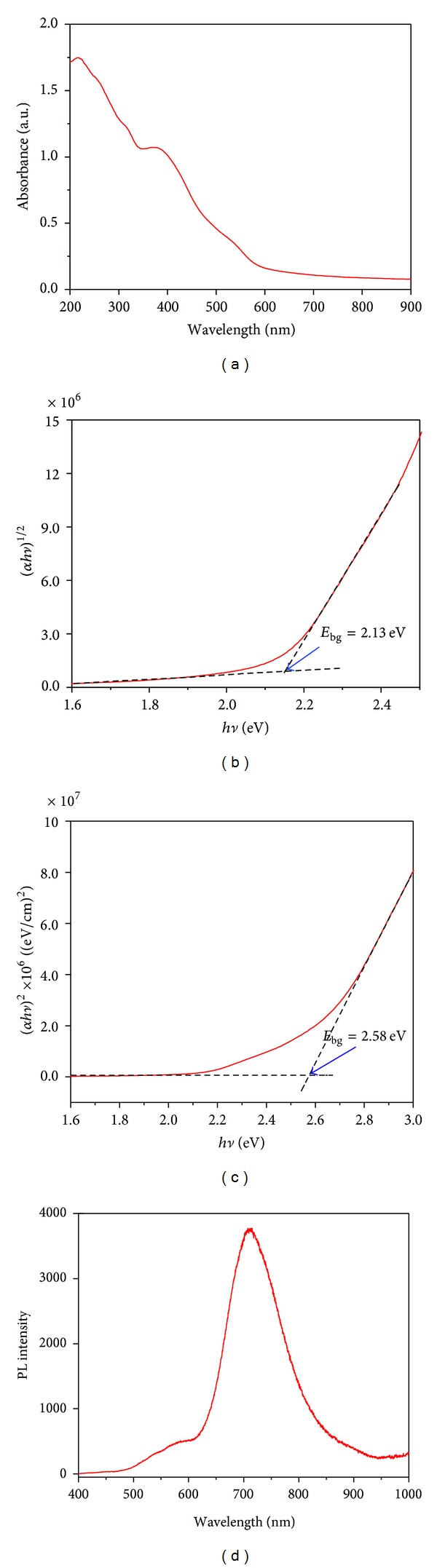
(a) UV-visible absorption spectrum of colloidal *α*-Fe_2_O_3_ nanoparticles. Plots of (b) (*αhν*)^2^ versus *hν* obtained due to direct transition and (c) (*αhν*)^1/2^ versus* hν* obtained due to indirect transition of *α*-Fe_2_O_3_ nanoparticles and (d) PL spectrum of colloidal *α*-Fe_2_O_3_ nanoparticles.

The UV-visible absorption spectrum for the colloidal *α*-Fe_2_O_3_ solution was recorded and is shown in Figure 1(a). The *α*-Fe_2_O_3_ nanoparticles exhibit strong absorption in the range of 200–400 nm in the UV region and weak absorption at 400–800 nm of the visible region. The two types of optical absorptions in the UV and visible regions are mainly attributed to the two kinds of electronic transition mechanisms. The former is due to the contribution of the direct charge transition of O_2_^−^2p→Fe^3+^ 3d (UV absorption), and the latter originates from the indirect charge transition of Fe^3+^ 3d→3d (visible absorption) [8–10]. The band-gap energy (*E*_bg_) value of the prepared *α*-Fe_2_O_3_ nanoparticles was derived from the relationship between the absorption coefficient and the incident photon energy of the *α*-Fe_2_O_3_ nanoparticles using Tauc's plot method [11, 12]. Consider

(1) $$\begin{array}{c} \alpha =\frac{A{\left(hv-E_{\text{bg}}\right)}^{n}}{hv}, \end{array}$$

where *α* is the absorption coefficient, *E*_bg_ is the band-gap energy, and *A* is the absorption constant. The absorption coefficient (*α*) was determined from the equation *α* = (2.303 × 10^3^) (*A*)/*L* by using the measured absorbance (*A*) and optical path length (*L*) (1 cm). The *n* is equal to 1/2 and 2 for allowed direct and allowed indirect transitions, respectively. By extrapolating the linear region in the plots of (*αhν*)^*n*^ versus *hν*, the band-gap energy values were obtained for the *α*-Fe_2_O_3_ nanoparticles (Figures 1(b) and 1(c)). The calculated band-gap energy values of 2.58 eV and 2.13 eV are due to the allowed direct transition and allowed indirect transition, respectively, for the *α*-Fe_2_O_3_ nanoparticles. This observed *E*_bg_ value is slightly higher and blue shifted compared to the results of previous reports because of the quantum size confinement in the nanoparticles. The photoluminescence spectrum of the *α*-Fe_2_O_3_ nanoparticles was recorded using excitation by 325 nm laser irradiation and is shown in Figure 1(d). The broad and intense PL peak at ~710 nm and shoulder peak at 590 nm are due to the band-edge emission of the *α*-Fe_2_O_3_ nanoparticles, which correspond to the band-gap energies of 1.75 and 2.1 eV for indirect and direct transitions.

### 3.2. XRD Analysis of *α*-Fe_2_O_3_ Nanoparticles

**Figure 2:**
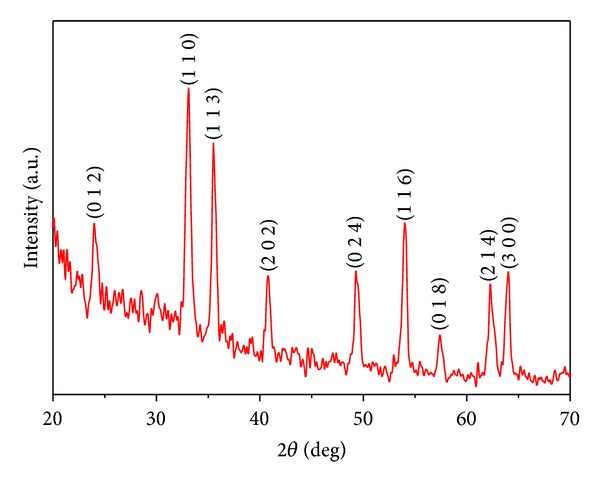
X-ray diffraction pattern observed for as-prepared *α*-Fe_2_O_3_ nanoparticles.

The typical X-ray diffraction peaks observed for the synthesized *α*-Fe_2_O_3_ nanoparticles (Figure 2) at 2*θ* values of 24°, 33°, 35°, 41°, 49°, 54°, 57°, 62°, and 64° correspond to the (0 1 2), (1 1 0), (1 1 3), (2 0 2), (0 2 4), (1 1 6), (0 1 8), (2 1 4), and (3 0 0) planes of the *α*-Fe_2_O_3_ nanoparticles, respectively [2, 9, 13]. The absence of diffraction peaks due to Fe(OH)_2_ and Fe(OH)_3_ indicates the complete transformation of these hydroxides to hematite during the hydrothermal process. The crystallite size was calculated to be ~40 nm for hematite particles using Scherrer's equation [13].

### 3.3. Morphological Studies of *α*-Fe_2_O_3_ Nanoparticles

**Figure 3:**
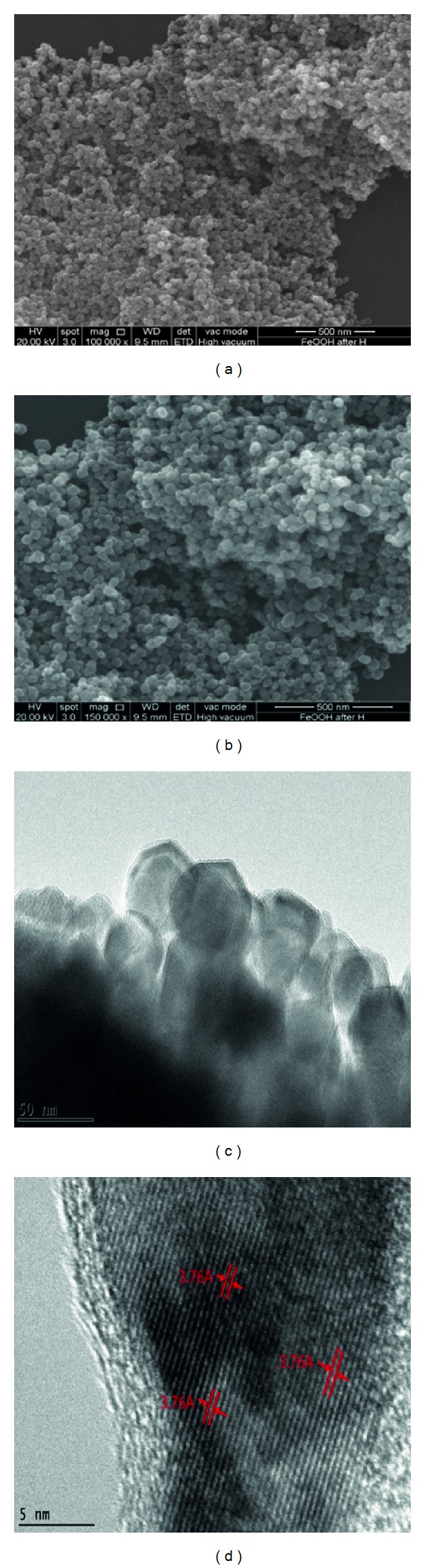
(a)-(b) FESEM and (c)-(d) HRTEM images of as-prepared *α*-Fe_2_O_3_ nanoparticles.

Figures 3(a) and 3(b) show FESEM images of the as-prepared *α*-Fe_2_O_3_ nanoparticles, which are composed of uniform monodispersed hematite nanoparticles. The corresponding TEM image (Figure 3(c)) further demonstrates that the obtained nanoparticles have a homogeneous size with a diameter of ~40 nm, which is in good agreement with that calculated from Scherrer's equation. The lattice resolved high-magnification TEM image (Figure 3(d)) shows a lattice fringe distance of approximately ~0.368 nm, which corresponds to the (0 1 2) plane of the hematite nanoparticles.

**Figure 4:**
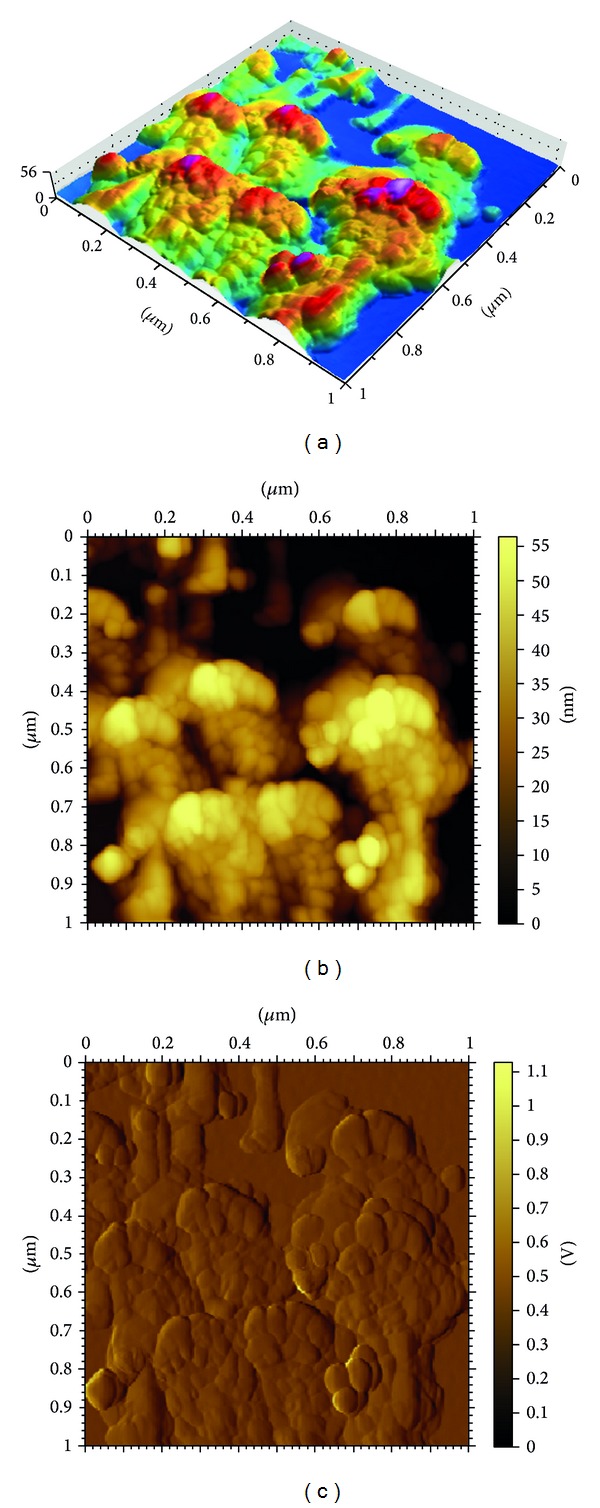
AFM topography observed for *α*-Fe_2_O_3_ nanoparticle-modified electrode in (a) angle view (3D), (b) top view (2D), and (c) amplitude view images.

In order to study the surface morphology of the *α*-Fe_2_O_3_ nanoparticle-modified electrode, AFM images were recorded, and their corresponding angle view (3D) and top view (2D) images are shown in Figure 4. The 3D AFM image of the *α*-Fe_2_O_3_ nanoparticle-modified electrode clearly shows the presence of nanoparticles on the surface of the electrode (Figure 4(a)). The 2D AFM image further confirms the presence of 30–50 nm *α*-Fe_2_O_3_ nanoparticles, and aggregated spherical particles are found on the surface (Figure 4(b)).

**Figure 5:**
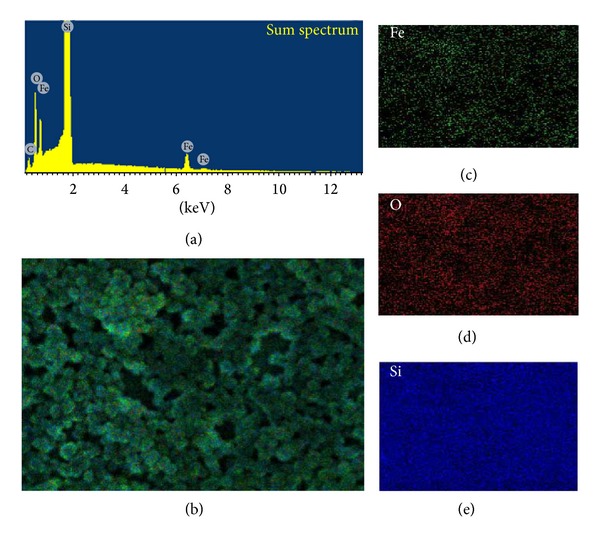
(a) EDX analysis and (b) FESEM-EDX elemental mapping of *α*-Fe_2_O_3_ nanoparticles on Si wafer for different elements: (c) Fe, (d) O, and (e) Si.

The EDX spectra and elemental mapping for the prepared *α*-Fe_2_O_3_ nanoparticles were recorded and are shown in Figure 5. The EDX elemental mapping from the FESEM analysis clearly confirms the presence of Fe and O in the *α*-Fe_2_O_3_ nanoparticles, along with Si as a result of the Si wafer. In the EDX spectrum, peaks are observed at 0.5, 6.4, and 7.1 for Fe, 0.2 and 0.4 for O, and 1.7 for Si because of the Si wafer. The absence of other elements indicates that the prepared nanoparticles have a high purity level.

### 3.4. Electrochemical Behavior of *α*-Fe_2_O_3_ Nanoparticle-Modified Electrode

**Figure 6:**
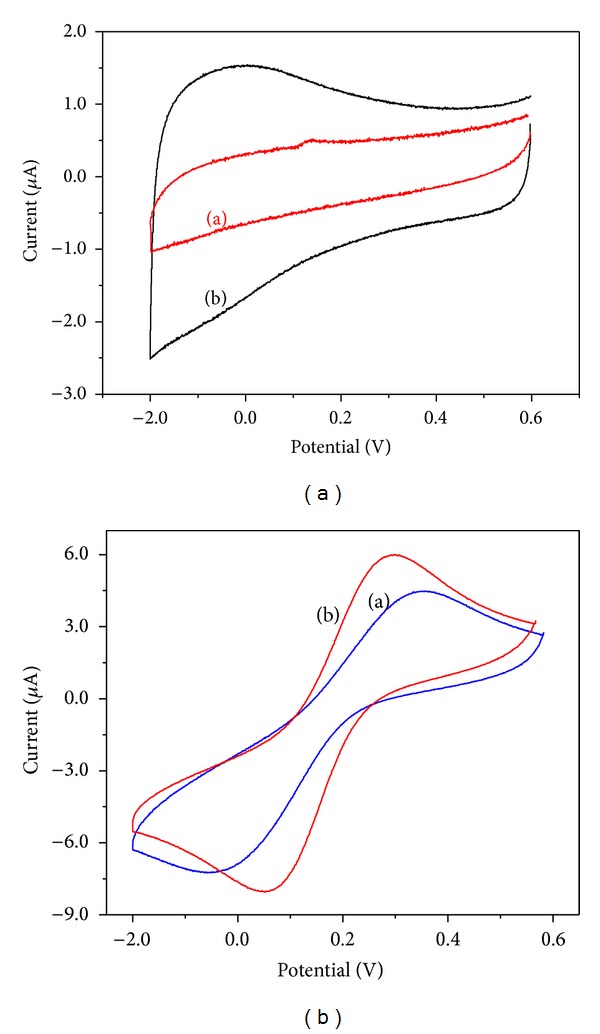
Cyclic voltammograms recorded for (a) bare GC and (b) *α*-Fe_2_O_3_ nanoparticle-modified electrode with 0.1 M KCl in (a) absence and (b) presence of 1 × 10^−3 ^M K_3_[Fe(CN)_6_] at scan rate of 50 mVs^−1^.

**Figure 7:**
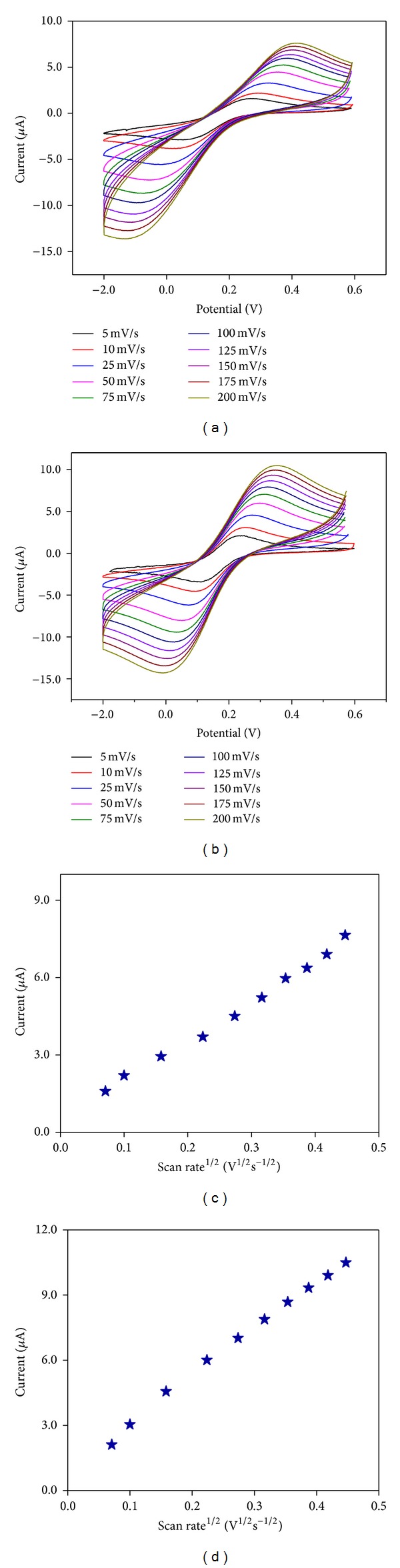
Cyclic voltammograms recorded at (a) bare GC and (b) *α*-Fe_2_O_3_ nanoparticle-modified electrode for 1 × 10^−3 ^M of K_3_[Fe(CN)_6_] in 0.1 M KCl at different scan rates: 5–200 mVs^−1^. The plots of the anodic peak current versus scan rate^1/2^ obtained for the (c) bare GC and (d) *α*-Fe_2_O_3_ nanoparticle-modified electrodes.

Cyclic voltammograms were recorded for thebare GC and *α*-Fe_2_O_3_ nanoparticle-modified electrode in 0.1 M KCl at a scan rate of 50 mVs^−1^ and are shown in Figure 6(a). It can be seen that the *α*-Fe_2_O_3_ nanoparticle-modified electrode has a higher current than the bare GCE, which is due to the presence of electroactive species (*α*-Fe_2_O_3_ nanoparticles) on the electrode surface. To study the electrochemical behavior of the *α*-Fe_2_O_3_ nanoparticle-modified electrode, an [Fe(CN)_6_]^3−/4−^ redox couple was used as an electrochemical probe. The cyclic voltammogram recorded for the [Fe(CN)_6_]^3−/4−^ couple at the bare GC electrode in a 0.1 M KCl solution showed a reversible electrochemical response for the [Fe(CN)6]^3−/4−^ couple (Figure 6(b)(a)). After the modification of the GC electrode with *α*-Fe_2_O_3_ nanoparticles, an increase in the peak currents and decrease in the peak potential separation (Δ*E*_*p*_) were observed for the [Fe(CN)_6_]^3−/4−^ couple (Figure 6(b)(b)) compared to those of the bare GC electrode. On the other hand, *α*-Fe_2_O_3_ nanoparticles can act as an electron transfer medium and enhance electron transfer during an electrochemical reaction. The introduction of *α*-Fe_2_O_3_ nanoparticles on the GC electrode surface facilitates the conduction pathway at the modified electrode surface. The electroactive surface area (*A*) of an electrode can be easily calculated from the slope obtained by the Randles-Sevcik plot described by the following:

(2) $$\begin{array}{c} i_{p}=2.69\times 10^{5}n^{3/2}v^{1/2}D^{1/2}AC, \end{array}$$

where *n*, *ν*, *D*, *A*, and *C* are the number of electrons, scan rate, diffusion coefficient (cm^2^s^−1^), surface area of the electrode, and concentration (in mol*·*L^−1^), respectively [14]. The diffusion coefficient (*D*) can be estimated using the Cottrell equation as follows:

(3) $$\begin{array}{c} I=\frac{{nFAD}^{1/2}C}{\pi^{1/2}t^{1/2}}, \end{array}$$

where *C*, *A*, *F*, and *n* are the bulk concentration (mol cm^−3^), area of the electrode (cm^2^), Faraday constant (96,485 Cmol^−1^), and number of electrons (*n* = 1 for [Fe(CN)_6_]^3−/4−^). By relating the amount of electroactive sites for each electrode in the [Fe(CN)6]^3−/4−^ solution that effectively transfers the charge to the species in solution, the electroactive surface area can be obtained. The surface areas for the modified electrode and bare GC were calculated to be 11.5 mm^2^ and 7.068 mm^2^, respectively, which show a 62.7% increase in the surface area of the modified electrode. An increase in the peak current resulted from increasing the electroactive surface area, as observed in the hematite-modified electrode [15]. The peak-to-peak separation (Δ*E*) of the CV results can be used to investigate the reversibility of the electrochemical process in the electrodes. The peak separation for a reversible process can be calculated as follows:

(4) $$\begin{array}{c} \Delta E_{p}=E_{\text{pa}}-E_{\text{pc}}=\frac{0.059}{n}, \end{array}$$

where Δ*E*_*p*_ is the minimum theoretical peak-to-peak separation, *E*_pa_ is the anodic peak potential, and *E*_pc_ is the cathodic peak potential. In a reversible reaction process, Δ*E* is greater than Δ*E*_*p*_, whereas in an irreversible reaction process, only one peak will be observed in one of the potential scans [14]. The Δ*E* values for the bare GCE and GC/*α*-Fe_2_O_3_-modified electrode were 291 and 237 mV, respectively, which are greater than the Δ*E*_*p*_ of 59 mV, indicating that the electrochemical redox reaction in both electrodes is quasireversible. The scan rate was varied in the range of 5–200 mVs^−1^ for the bare GC and GC/*α*-Fe_2_O_3_ electrodes with 1 × 10^−3 ^M of K_3_[Fe(CN)_6_] in 0.1 M KCl, and the results are shown in Figure 7.

**Figure 8:**
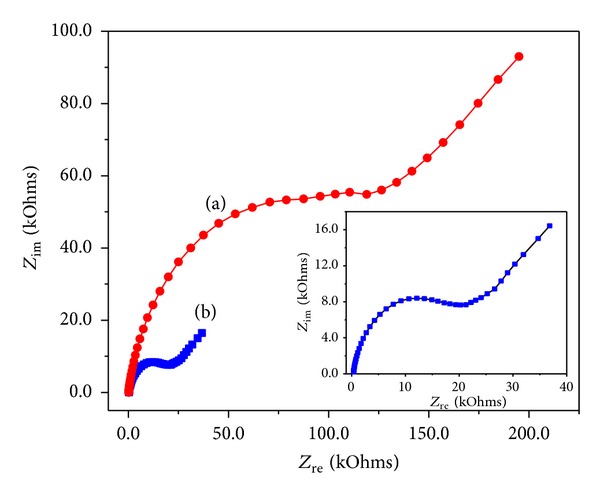
Electrochemical impedance spectra (EIS) obtained for (a) bare GC and (b) *α*-Fe_2_O_3_ nanoparticle-modified electrode in 1 × 10^−3 ^M K_3_[Fe(CN)_6_] in 0.1 M KCl. (*Inset*) Expanded view of EIS of *α*-Fe_2_O_3_ nanoparticle-modified electrode.

In order to investigate the electrical conductivities of the bare GC and *α*-Fe_2_O_3_ nanoparticle-modified electrode, electrochemical impedance spectroscopic (EIS) analyses were performed by dipping the bare GC and GC/*α*-Fe_2_O_3_ electrode into a solution containing 1 × 10^−3 ^M K_3_[Fe(CN)_6_] in 0.1 M KCl and using scanning frequencies ranging between 0.01 and 100,000 Hz. The observed EIS results for the bare GC and GC/*α*-Fe_2_O_3_ electrode are shown in Figure 8. The observed diameter of the semicircle is equal to the charge transfer kinetics of the redox probe at the electrode interface, where *Z*_re_ and *Z*_im_ are real and imaginary parts of the impedance spectra (*Z*). The bare GC electrode shows a semicircle with a larger diameter. Interestingly, the GC electrode modified with *α*-Fe_2_O_3_ nanoparticles showed a significant decrease in the semicircle diameter of the Nyquist plot. This indicated that the charge transfer resistance decreased upon the modification of the *α*-Fe_2_O_3_ nanoparticles. The charge transfer resistance (*R*_ct_) values for both the bare GC and the *α*-Fe_2_O_3_ nanoparticle-modified electrode were calculated by applying the following equation [16]:

(5) $$\begin{array}{c} R_{\text{ct}}=\frac{RT}{nFi_{0}}, \end{array}$$

where *R*_ct_ is the charge transfer resistance due to the transferring of electrons at the electrode/electrolyte solution interface, *R* is a gas constant, *T* is the experimental temperature, *n* is the electron transfer number of the redox process, which could be considered to be equal to one for [Fe(CN)_6_]^4−^/[Fe(CN)_6_]^3−^ couple, *F* is the Faraday constant, and *i*_0_ is the exchange current of the redox probe [16]. The bare GC and *α*-Fe_2_O_3_ nanoparticle-modified electrode had *R*_ct_ values of 180 and 25 kΩ, respectively. It should be noted that the *R*_ct_ value of the *α*-Fe_2_O_3_ nanoparticle-modified electrode is lower than that of the bare GC electrode, which is mainly attributed to the high electron conductivity of the *α*-Fe_2_O_3_ nanoparticle-modified electrode and the sluggish electron transfer behavior of the bare GC. This clearly shows that the *α*-Fe_2_O_3_ nanoparticle-modified electrode is a promising candidate for electrocatalysis and sensor applications.

### 3.5. Electrocatalytic Activity of *α*-Fe_2_O_3_ Nanoparticle-Modified Electrode toward Dopamine

**Figure 9:**
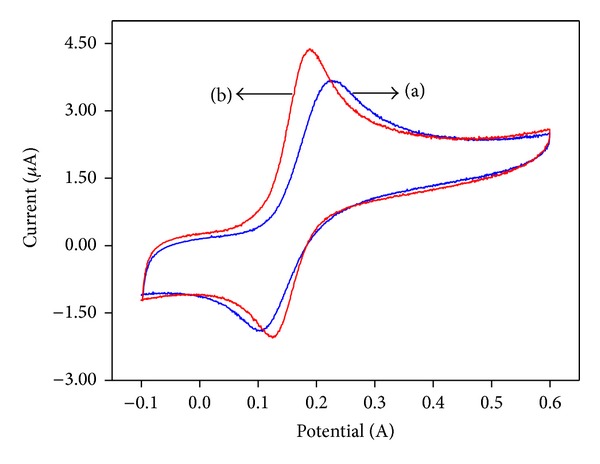
Cyclic voltammograms recorded at (a) bare GC and (b) *α*-Fe_2_O_3_-modified electrode for 50 *μ*M of DA in 0.1 M PBS solution (pH = 6.8) at scan rate of 50 mVs^−1^.

Cyclic voltammograms were recorded from the bare GC and GC/*α*-Fe_2_O_3_-modified electrode for 50 *μ*M of DA in a 0.1 M PBS solution (pH = 6.8) at a scan rate of 50 mVs^−1^ and are shown in Figure 9. It can be observed that the bare GCE has quasireversible, poorly defined redox peaks and a low electrochemical response toward DA. However, a significant increase in the redox peak current was detected for the GC/*α*-Fe_2_O_3_-modified electrode, contributing to its improved conductivity performance. Hence, the GC/*α*-Fe_2_O_3_-modified electrode exhibited reversible behavior and possessed faster electron transfer kinetics, as reflected in its low Δ*E*_*p*_ value.

**Figure 10:**
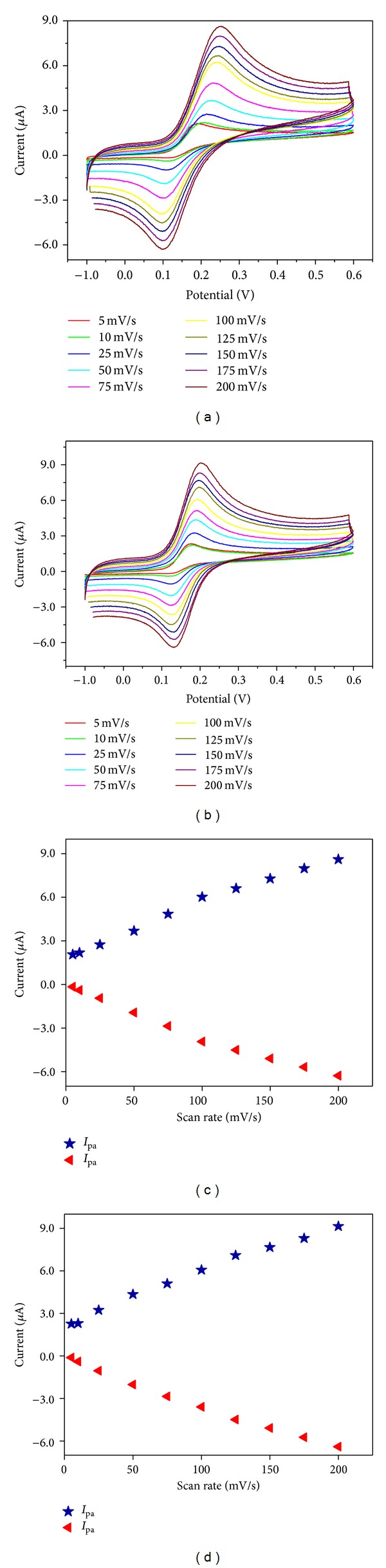
Cyclic voltammograms recorded for (a) bare GC and (b) *α*-Fe_2_O_3_ nanoparticle-modified electrode with 50 *μ*M of DA in 0.1 M PBS (pH = 6.8) at different scan rates: 5–200 mVs^−1^. The plots of the peak current versus scan rate^1/2^ obtained for the (c) bare GC and (d) *α*-Fe_2_O_3_ nanoparticle-modified electrode.

The effect of the scan rate on the DA oxidation was studied for the bare GC and GC/*α*-Fe_2_O_3_-modified electrode by varying the scan rate from 5 to 200 mVs^−1^ with 50 *μ*M of DA in a 0.1 M PBS (pH = 6.8) (Figure 10). At the 200 mVs^−1^ scan rate, we observed a redox peak with an anodic-to-cathodic ratio of 1.37, which was slightly higher than the unit value expected for the ideal voltammetric behavior of a GC/*α*-Fe_2_O_3_-modified electrode for reversibility. The peak separations (Δ*E*) of the bare electrode and GC/*α*-Fe_2_O_3_-modified electrode for all the scan rates in the range of 5–200 mVs^−1^ were greater than 295 mV (due to the two electron transfers), which indicated a reversible redox process. Increasing the scan rate increased the peak separation (Δ*E*) because of the chemical interaction between the DA and the modified electrode. Simultaneous increases in the anodic and cathodic current intensities were also observed when increasing the scan rate. A plot of the anodic and cathodic current (*I*_pa_) peaks versus the square root of the scan rate is shown in Figure 10. It shows the linear relation for the GC/*α*-Fe_2_O_3_-modified electrode (*R* ^2^ = 0.9943 and 0.9931), which indicates a diffusion controlled redox process [3, 17–20].

### 3.6. Influence of pH on Electrocatalytic Activity of *α*-Fe_2_O_3_ Nanoparticle-Modified Electrode

**Figure 11:**
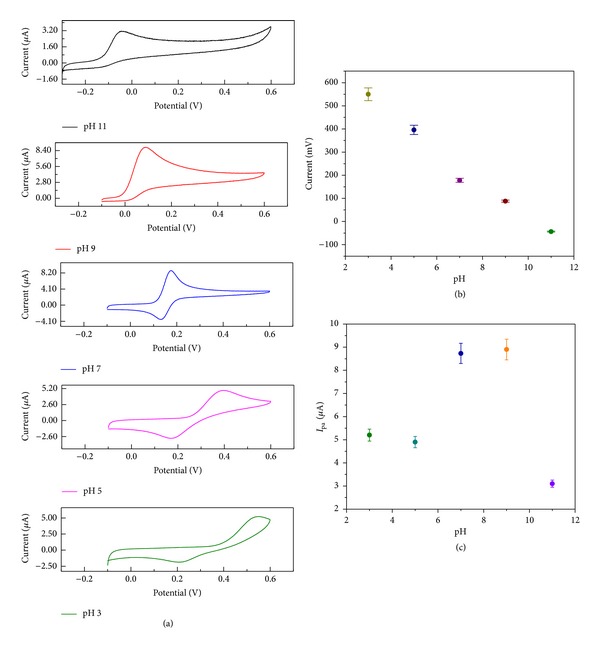
(a) Cyclic voltammograms recorded at *α*-Fe_2_O_3_ nanoparticle-modified electrode for 50 *μ*M of DA in 0.1 M PBS with different pH values at scan rate of 50 mVs^−1^. Plots of (b) anodic peak potential (*E*_pa_) and (c) anodic peak current (*I*_pa_) against different pH values for PBS solution.

The influence of the electrolyte solution pH on the electrochemical reaction to the DA in the presence of the *α*-Fe_2_O_3_ nanoparticle-modified electrodewas studied in phosphate buffer solutions with various pH values ranging from 3 to 11, and the observed cyclic voltammograms are shown in Figure 11(a). It can be observed that increasing the pH of the electrolyte solution leads to negative shifts in both the anodic and the cathodic peak potentials, which suggests that the redox reaction of DA includes some protons transfers at the modified electrode. In addition, the influence of the pH on the anodic peak potential of DA was also studied, and the obtained results are shown in Figure 11(b). From Figure 11(c), it can be noted that the applied potential (*E*_pa_) versus the pH shows a linear relationship in the pH range of 3 to 11, with a slope value of −75.05 mV/pH and this observed slope value is higher than the theoretical value of −59 mV/pH.

### 3.7. Chronoamperometric Detection of Dopamine

**Figure 12:**
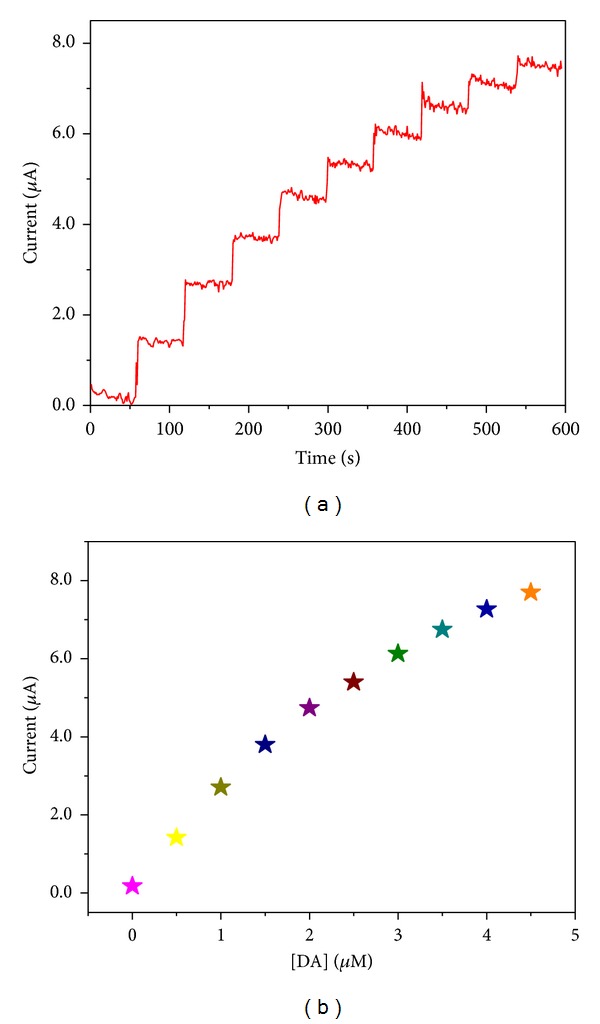
(a) Chronoamperometric I-T curve obtained for *α*-Fe_2_O_3_ nanoparticle-modified electrode during successive addition of 0.5 *μ*M of DA in 0.1 M PBS (pH = 6.8) at applied potential of 200 mV. (b) The calibration curve obtained upon the 0.5 *μ*M addition of DA in 0.1 M PBS (pH = 6.8).

The chronoamperometric technique was also used to determine the applicability of the GC/*α*-Fe_2_O_3_-modified electrode for DA sensing.  Figure 12 shows the chronoamperometric response of the GCE/*α*-Fe_2_O_3_-modified electrode upon the stepwise addition of 500 nM into the 0.1 M PBS (pH = 6.8) at an applied potential of 200 mV.  Figure 12 shows the increase in the current with increasing DA concentration in the range of 0.5 to 5 *μ*M. From the calibration plot (Figure 12(b)), it is observed that increasing the DA concentration leads to slow saturation of the current response due to the saturation of the catalytic sites of the modified electrode at a high concentration of DA.

The linear relation of the dopamine concentration and the current in the range of 0 to 2 *μ*M can be calculated from a calibration plot with a correlation coefficient (*R*) of 0.9845 (*n* = 10) for the following regression equation [21]: *I* (*μ*A) = 0.8867 + 1.6546 (*μ*M). The limit of detection (LoD) was calculated to be 1.6 *μ*M (signal-to-noise ratio (*S*/*N*) = 3) by substituting the blank standard deviation (*σ* = 0.8867) and sensitivity (*m* = 1.6546 *μ*A/*μ*M) in the 3σ/m criterion [22]. The response time for each DA addition was observed to be ~3 s, which indicates a fast electron transfer with this modified electrode.

### 3.8. Square Wave Voltammetric Detection of Dopamine

**Figure 13:**
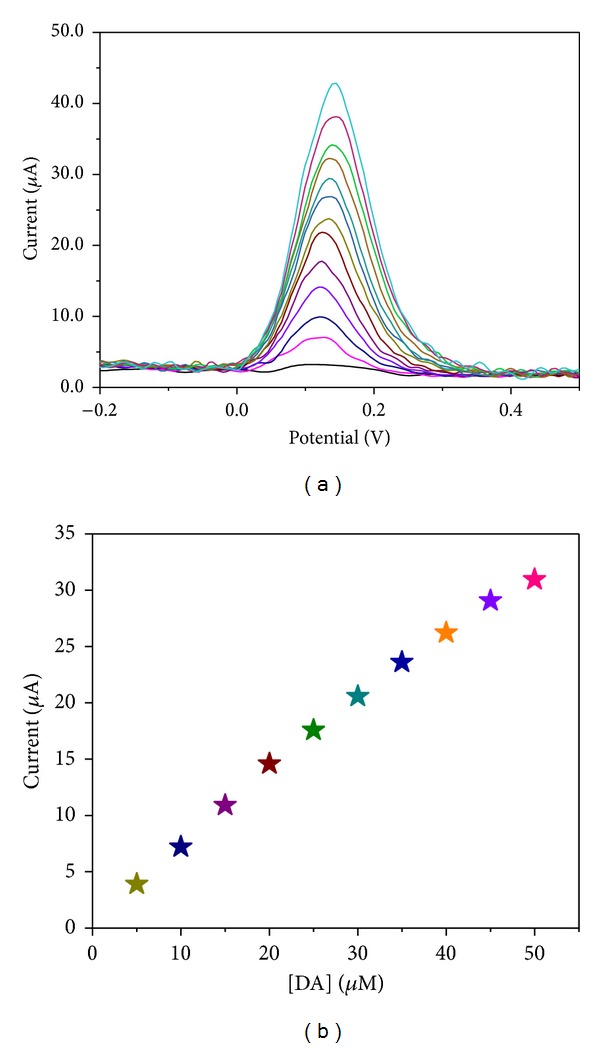
(a) Square wave voltammograms recorded for *α*-Fe_2_O_3_ nanoparticle-modified electrode during successive addition of 5 *μ*M of DA in 0.1 M PBS (pH = 6.8) at scan rate of 100 mVs^−1^. (b) The calibration curve obtained upon the 5 *μ*M addition of DA in 0.1 M PBS (pH = 6.8).

The influence of the dopamine concentration on the electrocatalytic activity was investigated with the GC/*α*-Fe_2_O_3_-modified electrode using a square wave voltammetric technique by adding different concentrations of DA into the 0.1 M PBS (pH = 6.8), and the results are shown in Figure 13. It is observed that when the DA concentration increases, the peak current also increases up to 90 *μ*M of DA. When the DA concentration was greater than 90 *μ*M, the modified electrode did not show any further increase in the peak current. This phenomenon is mainly attributed to the saturation of the catalytic sites of the modified electrode. The calibration plot obtained for the modified electrode current response against the dopamine concentration is shown in Figure 13, and it shows a linear relationship toward the DA concentration in the range of 0.0 to 90 *μ*M. A linear relationship with a correlation coefficient (*R*) of 0.9974 (*n* = 10) for the regression equation *I* (*μ*A) = 0.048 + 1.6827 (*μ*M) was obtained [21]. The LOD was found to be 236 nM (*S*/*N* = 3) by substituting the blank standard deviation (*σ* = 0.048) and sensitivity (*m* = 0.6099 *μ*A/*μ*M) in the 3σ/m criterion [22].

### 3.9. Selectivity Study of *α*-Fe_2_O_3_ Nanoparticle-Modified Electrode

**Figure 14:**
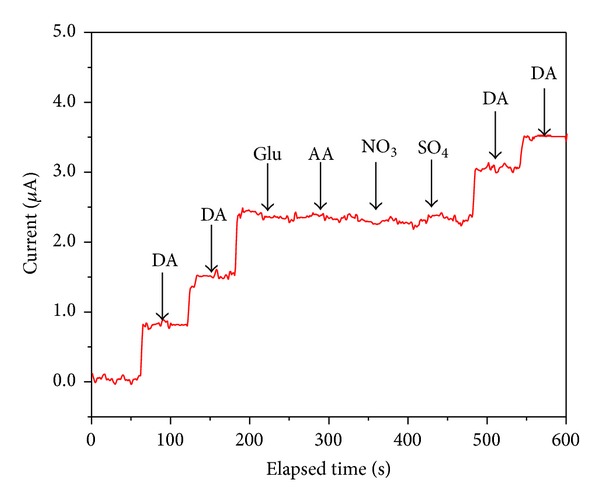
Chronoamperometric curve obtained for hematite-modified electrode upon addition of DA and interference sources, including ascorbic acid, glucose, sulphate, and nitrate, at applied potential of 200 mV.

In order to study the selectivity of the *α*-Fe_2_O_3_ nanoparticle-modified electrode toward DA, chronoamperometric curves were recorded with common interference sources such as ascorbic acid, glucose, sulphate, and nitrate and are shown in Figure 14. It can be seen that upon the addition of DA, the current is increased, whereas when 100-fold higher concentration of an interference source is added, such as AA, Glucose (Glu), SO_4_^−^, or NO_2_^−^, there is no obvious increase in the current. Further, when DA is added to the same solution, the current increases. This clearly suggests that the fabricated *α*-Fe_2_O_3_ nanoparticle-modified electrode is more selective toward DA.

## 4. Conclusion

Hematite (*α*-Fe_2_O_3_) nanoparticles were synthesized using a hydrothermal method and characterized by UV-vis, PL, XRD, TEM, AFM, FESEM, and EDX analyses. The experimental results indicated the formation of uniform hematite nanoparticles with an average size of 45 nm. The so-formed *α*-Fe_2_O_3_ nanoparticles were used to fabricate a sensor electrode for the detection of DA. The electrochemical behavior of this GC/*α*-Fe_2_O_3_ electrode was studied by using CV and EIS techniques with an electrochemical probe, an [Fe(CN)_6_]^3−/4−^ redox couple. The electrocatalytic activity was investigated toward DA oxidation in a phosphate buffer solution (pH 6.8) by varying different experimental parameters. The chronoamperometric study showed a linear response in the range of 0–2 *μ*M with LoD of 1.6 *μ*M for DA. The square wave voltammetry showed a linear response in the range of 0–35 *μ*M with LoD of 236 nM for DA. In a biological system containing a micromolar concentration of DA [23], submicromolar detection would be beneficial using an SWV and CA method for a modified electrode. The results of this study suggested that *α*-Fe_2_O_3_ nanoparticles could be a promising candidate for the fabrication of ultramicroelectrodes for in vivo DA detection applications.
